# A comparative study of multiple clinical enterovirus 71 isolates and evaluation of cross protection of inactivated vaccine strain FY-23 K-B in vitro

**DOI:** 10.1186/s12985-017-0872-8

**Published:** 2017-10-26

**Authors:** Ting Yang, Hua Li, Lei Yue, Xia Song, Tianhong Xie, Shaohui Ma, Huaqing Meng, Ye Zhang, Xin He, Runxiang Long, Rong Yang, Fangyu Luo, Zhongping Xie, Qihan Li

**Affiliations:** Institute of Medical Biology, Chinese Academy of Medical Sciences and Peking Union Medical College, Kunming, 650118 China

**Keywords:** Hand, Foot and mouth disease, Enterovirus 71, Cross protection, Genetic conditions

## Abstract

**Background:**

Enterovirus 71 (EV71) is one of the causative agents of hand, foot and mouth disease, which mostly affects infants and children and leads to severe neurological diseases. Vaccination offers the best option for disease control. We have screened the virus strain FY-23 K-B, which is used as an inactivated vaccine strain. An important issue in the development of vaccines is whether they provide cross protection against all other strains.

**Methods:**

We collected and identified 19 clinical EV71 isolates from mainland China, which all belong to the C4 genotype. We established growth curves of the strains in Vero cells, performed genetic analysis, and evaluated the cross protection efficacy through neutralizing assays using antisera from a rabbit, monkey and adult human immunized with the FY-23 K-B vaccine strain.

**Results:**

The antisera showed broad cross protection among the C4 subgroup strains and homotype strain. Neutralizing indexes (NIs) among the isolates and homotype strain of antisera varied between 56.2–1995.3 for rabbit, 17.8–42,169.7 for monkey and 31.6–17,782.8 for human, whereas NIs against Coxsackievirus A16 or other enteroviruses were below 10.

**Conclusions:**

These results suggested that FY-23 K-B used as an antigen could elicit broad spectrum neutralizing antibodies with cross protective efficacy among C4 genotype strains.

## Background

Enterovirus 71 (EV71) is a small, single-stranded, positive-sense RNA virus that belongs to the family *Picornaviridae* of the genus *Enterovirus* [1]. EV71 and Coxsackievirus A16 (CA16) are recognized as the two most important etiological agents of hand, foot and mouth disease (HFMD), and cause a wide range of clinical manifestations, including cutaneous, visceral and neurological diseases [1]. Morbidity and mortality is high for HFMD in Southeastern Asian countries including Singapore [2], South Korea [3], Malaysia [4], Japan [5], Vietnam [6], mainland China and Taiwan [7, 8]. HFMD is generally a self-limiting disease [9], but sometimes causes severe neurological diseases such as aseptic meningitis, acute flaccid paralysis, and brainstem encephalitis. Although CA16 and some other enteroviruses significantly contribute to morbidity of HFMD, the overwhelming majority of reported severe cases are attributed to infection by EV71. Epidemiological data show that HFMD due to infection by EV71 occurs most frequently in children aged <5 years.

HFMD was classified as a Category C notifiable infectious disease by the National Health and Family Planning Commission of the People’s Republic of China on May 2, 2008, and since then, cases of HFMD infection and mortality have been well documented. The data show a serious health issue in China, highlighting the urgent need for effectively controlling the disease through public health management. Recent extensive efforts have been made to develop vaccines against EV71 including inactivated, attenuated, recombinant subunit, virus-like particle, and DNA vaccines. The accumulated data show that the inactivated vaccine is the most feasible, safe, and efficacious.

Three clinical trials of inactivated EV71 vaccines are being conducted in mainland China. FY-23 K-B, a clinical EV71 isolate from an HFMD outbreak in China, was identified and propagated for the development of an inactivated vaccine at our institute. The vaccine strain, FY-23 K-B, exhibited excellent biological activity, genetic stability and immunogenicity in our studies with rhesus monkeys [10]. On December 3, 2015, the first inactivated EV71 whole-virus vaccine developed by the Institute of Medical Biology, Chinese Academy of Medical Science (CAMS) was approved by the China Food and Drug Administration. The vaccine showed good safety and protective efficacy in clinical trials [11, 12]. In January 2016, another inactivated EV71 vaccine from Sinovac Biotech Co. (Beijing) was approved for marketing in China [13]. The third inactivated vaccine in the mainland of China which was made by Vigoo Biological Co.(Beijing), was licensed at the end of 2016 [14].

Molecular epidemiological investigations suggest that circulating subgroups vary among areas and shifts in subgroup dominance are common. Thus, an ideal vaccine strain must provide effective cross protection against variable clinical isolates [12, 15]. However, to date, the crossprotective activity of the EV71 vaccine stains is less clear. To further our knowledge of vaccine efficacy, 19 strains isolated clinically from different areas in China were used to assess the cross protective efficacy of the vaccine through in vitro neutralization assays using antisera from a rabbit, a monkey and an adult human, immunized with vaccine strain FY-23 K-B.

## Methods

### Cells and viruses

EV71 strain FY-23 K-B, isolated from an HFMD outbreak in Fuyang, China in 2008, was used to develop an inactivated vaccine. The vaccine is currently available in China. All virus strains used, including the vaccine strain FY-23 K-B, a homotype A strain BrCr, and nineteen clinical isolates from different areas in China, and six other enterovirus strains including PolioI, PolioII, Echo2, Echo6, Coxsackievirus A7, and Coxsackievirus B5, were collected and generously provided by the Department of Viral Immunology, Institute of Medical Biology, CAMS. Coxsackievirus A16 was clinically isolated and identified in our lab. Viruses were isolated and propagated in Vero cells, kindly donated by the Dutch National Institute for Public Health and the Environment, and maintained at the Department of Quality Control, Institute of Medical Biology, CAMS. Vero cells were cultured using Eagle’s minimum essential medium (Sigma, St. Louis, MO, USA) containing 5% fetal bovine serum (Gibco, Gaithersburg, MD, USA).

### Antisera

A rhesus monkey (5-month-old male, 1.2 kg) and a New Zealand rabbit (6-month-old female, 2.6 kg) were immunized subcutaneously with inactivated vaccine prepared with FY-23 K-B as previously described [10]. Sera were collected at the endpoint. Human serum was obtained from an adult inoculated with inactivated EV71 vaccine.

### Virus titration by CCID50 assay and growth kinetics of EV71 strains

Virus titers were determined as described previously [16, 17], based on typical cytopathic effect (CPE) developing in infected Vero cells and expressed as 50% cell culture infective dose (CCID_50_). Monolayer Vero cells in a 96-well microplate were inoculated with 100 μL 10-fold serial dilutions of EV71 virus, and cultured at 37 °C for 7 days. CPE was observed daily, and CCID_50_ was calculated using the Bethrens-Kärber method.

### Sequence analysis for EV71 VP1

Viral RNA was extracted from previously prepared virus stocks using AxyPrep Body Fluid Viral DNA/RNA Miniprep Kit (Axygen, CA, USA). Reverse transcriptase polymerase chain reaction (RT-PCR) was conducted with One-Step RT-PCR kits (Takara, Dalian, China) according to the manufacturer’s instructions with reverse transcription at 50 °C for 30 min. PCR was 94 °C for 2 min followed by 40 cycles of 94 °C for 30 s, 60 °C for 30 s, and 72 °C for 1 min. EV71 VP1-specific primers were designed according to the sequence of the strain FuyangAnhuiPRC (GeneBank accession no.EU703812.1) as follows: forward (F):AAGGATGCTAGTGATATCCT; and reverse (R): CATTGTGAGTGGCAAGAT. DNA sequencing was carried out on an automatic DNA sequencer (Takara Biotech). Phylogenetic and molecular evolutionary analyses were conducted by using MEGA version5.10.

The reported nucleotide acid sequences were deposited in the GeneBank database (Table1).

### Neutralization assays for collected antisera

Antisera samples were heat inactivated at 56 °C for 20 min before use. Mixed with 50μL of two-fold serially diluted antisera were equal volumes of medium containing 100 CCID_50_ EV71 in 96-well plates. Samples were incubated at 37 °C for 1.5 h to facilitate antibody binding to viruses. A 100 μL Vero cell suspension was added to a cell density 10^5^/mL. After culturing at 37 °C for 7 days, cells were observed for CPE. The highest dilution of serum that protected at least half of cells in one well from CPE was designated the neutralization titer.

### Determination of neutralization index

Viruses serially diluted 10-fold were mixed with an equal volume of medium containing 4 neutralizing unit (NU) antisera as the experimental group, and mixed with only medium as the control group. Experimental procedures were as described in the section ‘Neutralization assays for collected antisera’. Data were expressed as an index value that suggested correlation between virus growth in serum compared to growth in cultures infected with virus alone. Neutralization index (NI) was calculated as the antilogarithm of the difference between control and antisera virus titers. Samples with a NI less than 10, indicating a 10-fold decrease in virus production, were considered non-neutralizing. Samples with NI between 10 and 50 were considered as questionably neutralizing. Samples with NI exceeding 50 were considered neutralizing.

### Statistical analysis

SPSS 22.0(SPSS, Chicago, IL, USA) was used for statistical analysis. Spearman’s test was employed to analyze bivariant correlations.NI values did not match Gaussian distributions so nonparametric tests were used. Phylogenetic trees were plotted using MEGA 5.1 software.

## Results

### Virus titers

**Fig. 1:**
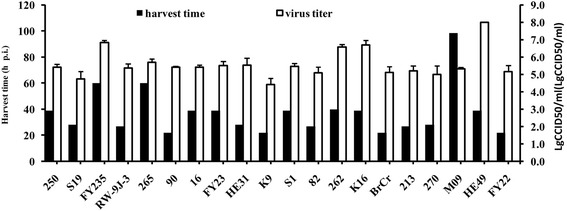
The Vero cells were respectively infected with twenty EV71 strains at an MOI of 2.0 and cells were harvested when the cytopathic effect exceeded 95 %, then the viruses were titrated by microplate cytopathic effect method. Virus titers were showed as MEAN ± SD (*n* = 3 independent experiments)

The nineteen isolates propagated well in Vero cells. At a Multiplicity of Infection (MOI) of 2.0, viruses were harvested between 22 h and 90 h post infection (Fig. 1), when the observed cytopathic effect exceeded 95%. Virus titers showed a significantly positive correlation with harvest time (Spearman’s correlation coefficient = 0.651, *P* = 0.002).

### One-step growth curves of twenty EV71 strains

To establish one-step growth curves for 20 EV71 strains in Vero cells, cells were infected with 10-fold serially diluted virus, CPE was observed daily and virus titer was calculated.

**Fig. 2:**
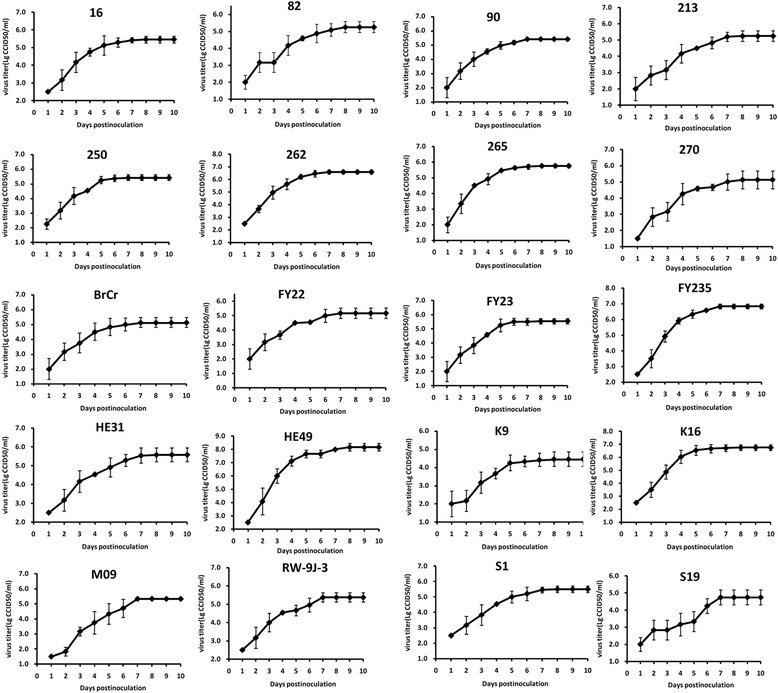
One-step growth curves of ninety clinical isolates and the prototype strain in Vero cells. The viruses were titrated by microplate cytopathic method. The data was shown as MEAN ± SD (*n* = 3 independent experiments)

The viral rapid increase phase differed among the 20 strains (Fig. 2). Almost all viruses entered eclipse phase before 24 h post infection (p.i.), and then reached a plateau between 4 and 7 days, and the average was 5.6 days.

### Phylogenetic analysis of VP1 region of EV71 isolates

RT-PCR and sequencing was used to identify 19 clinical isolates. Phylogenetic analysis of the isolates was based on the alignment of complete VP1 nucleotide sequences by the neighbor-joining method. Genogroups and subgenogroups were determined by comparing sequences to reference strains in GenBank (Table 1).

**Table 1:** VP1 gene nucleotide sequences of the HEV71 strains used to generate the EV71 phylogenetic dendrogram

Strain	GenBank No.	Strain	GenBank No.
JN200803	JF913464	03KOR00	DQ341356
JN200804	HQ825317	06KOR00	DQ341355
Zhejiang08	EU864507	BrCr	U22521
F1CHN00	AB115490	6910OK87	AF135901
SHZH98	AF302996	Nagoya	AB059813
6FAUS699	AF376107	3799SIN98	DQ341354a
7FAUS699	DQ341357	MY1049SAR97	DQ341368
1MAUS1200	DQ341361	S19841SAR03	AY258310
S100862SAR98	AF376080	PP37MAL01	DQ341365
SB2864SAR00	DQ341366	MS742387	U22522
CA16 G10	U05876		

**Fig. 3:**
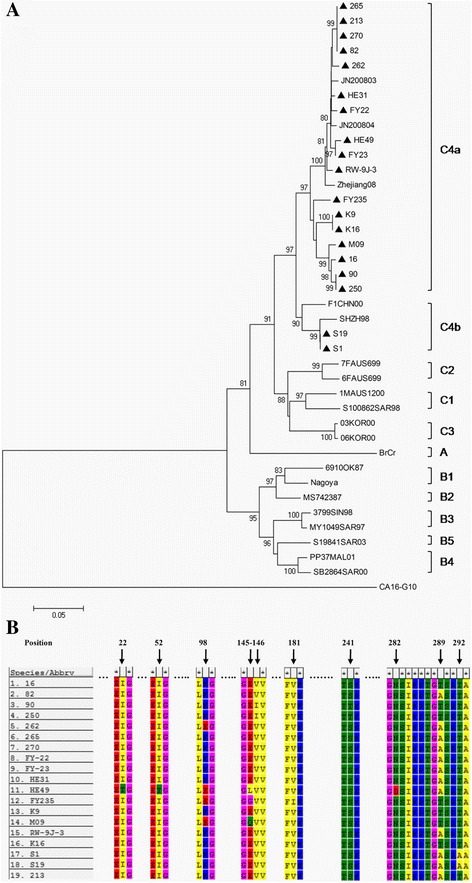
Molecular character analysis of EV71 isolates. **a** Phylogenetic analysis based on nucleotide sequences of EV71 VP1 (891 bp). ▲Indicates EV71 strains using in the current study. **b** Amino acid mutations of the ninety EV71 strains in VP1 region

EV71 strains isolated in mainland China were closely related to each other and grouped into genotype C, forming a new genetic lineage (C4). Consistent with previous studies [15, 18], the 19 clinical isolates clustered in genotype C4 in the phylogenetic tree (Fig. 3a).Nucleotide identities between 93.3%–100%.

To identify the outcomes of genetic changes, the deduced amino acid sequences of VP1 were compared among the 19 isolates. Nucleotide differences in VP1 led to some amino acid changes. Positions 22, 52, 98, 145, 146, 181, 241, 282, 289 and 292 in the amino acid sequence were mutated (Fig. 3b).

### Neutralization indexes of vaccine strain antisera using twenty EV71 strains

**Fig. 4:**
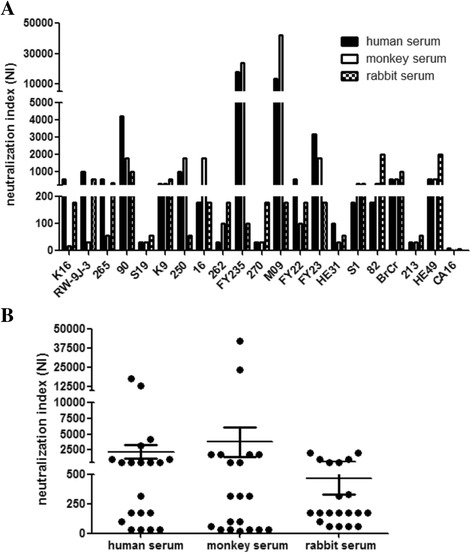
NI values of antisera collected from human, monkey and rabbit measured using different EV71 strains. **a** NIs assessed using ninety EV71 clinical isolates and the prototype strain. **b** NIs determined using antisera from different species. Data was shown as MEAN ± SEM (*n* = 20)

Antisera with EV71-neutralizing titer 1:4(4NU) from a human, monkey and rabbit immunized with vaccine strain FY23K–B, exhibited broad and effective neutralization activity across all isolates (Fig. 4). Antisera showed significantly different neutralization (*P* = 0.02) among clinical isolates. NIs for the 20 strains varied with 32–17,783 for human serum, 18–42,170 for monkey and 56–1995 for rabbit (Fig. 4b).No significant differences were observed among the antisera from human, monkey or rabbit (*P* = 0.655).

The NI of antisera with 4EU used against eight strains of other enteroviruses was <10(data not shown). This demonstrated that antisera from the EV71 vaccine strain provided no cross protection against other enteroviruses, including CA16.

## Discussion

Potent immunogenicity and broad-spectrum cross protection are crucial for development of an effective vaccine [19]. In this study, using 19 EV71 clinical isolates collected from different areas in China and eight enterovirus strains other than EV71, we evaluated the cross protective efficacy of antisera against the vaccine strain. The results furthered our knowledge of the clinical application of the current vaccine.

The vaccine strains currently used for the development of the inactivated vaccine in China all belong to the C4a cluster of subgenotype C4. Nucleotide sequence homology among the six vaccine candidates was 93.3%–99.7%. EV71 strains of genotypes A, B and C shared 94.0% amino acid sequence homology [12, 15]. Lee et al. reported antigenic differences between different genogroups but not among different genotypes belonging to the same genogroup [20].

This study showed that the inactivated EV71 vaccine produced with FY-23 K-B strain exhibited broad neutralization activity against 19 clinical isolates from mainland China and the prototype EV71 BrCr strain. This result was consistent with observations in a phase III clinical trial of the inactivated EV71 vaccine [15] which indicated that the vaccine is highly effective for controlling of EV71-associated HFMD. Our results suggested the vaccine does not protect against CA16 (NI value was below 10), another important causative agent of HFMD. This result suggests, in the context of success of the EV71 vaccine, the development of a CA16 vaccine is increasingly urgent for prevention of HFMD. In clinical trials, 1:8 is the threshold for EV71-neutralizing antibodies for evaluation of vaccine efficacy [12]. In our study, antisera with an EV71-neutralizing titer of 1:4(4NU) were broadly effective in vitro. Antisera from different species had slightly different crossprotective efficiency (Fig. 4b).

The antisera provide stronger neutralizing protection against the parental strain than against the same or other genotype strains is expected because of sequence homology. In studies from Arita et al. [21] and Li et al. [22], anti-EV71 serum from different species showed the highest neutralization activity against the homotype strain. However, in our study, the NI of the antisera was not highest against the parental strain FY-23. Furthermore, the results suggested that which one of the virus strains would be chose in the neutralization assays which are used for the surveillance of virus infection or evaluation of vaccine efficacy was adequately considered.

EV71 VP1–4 constitutes the viral capsid particle. VP1 is the major capsid protein and contains antigenic epitopes eliciting protective neutralization antibodies [19]. However, the neutralizing epitopes of VP1 have not been fully identified. Peptides containing amino acids 163–177 or 208–222 of EV71 VP1 are capable of eliciting neutralizing antibodies [20]. In our study, no amino acid mutations were found in neutralizing antigenic peptides reported among the 19 clinical isolates. This result supported the suggestion that effective cross protection against variable clinical virus strains can be obtained by vaccination with strain FY23K–B because of their relatively conservative antigenic epitopes in the EV71 capsid protein. However, NIs of antisera from human and monkey against FY235 and M09 were much higher than against other strains. The two strains share three common changes of amino acids in the VP1 region, located at position 98(K to E), position 145(E to G or C) and position 289(A to T). Compared to the parental strain, the FY-23, and FY-23 K-B strains had two amino acid substitutions in theVP1 region; at position 226 and position 282 [10]. Further study is required to determine whether these sites are involved in eliciting neutralizing antibodies.

## Conclusions

The inactivated EV71 vaccine prepared from FY-23 K-B which shows good antibody responses and effective protection against HFMD has been licensed. We demonstrate here the cross protection of this vaccine among different EV71 isolates. We also show its limited effectiveness against CA16 infection. Thus it may more urgent to develop a CA16 vaccine for full prevention of HFMD. In addition, the neutralization assay for determining antibody titer is common in quantifying the efficacy of a vaccine. We strongly suggest that the choice of strain chosen for the titration be carefully considered, as the antibody titers titrated by different strains are dissimilar, probably due to the mutations within the VP1 region of the viral genome.

## Abbreviations

CA16: Coxsackievirus A16
CAMS: Chinese Academy of Medical Science
CCID_50_: 50% Cell culture infective dose
CPE: Typical cytopathic effect
EV71: Enterovirus 71
HFMD: Hand, foot and mouth disease
MOI: Multiplicity of Infection
NI: Neutralization index
NU: Neutralizing Unit
PCR: Polymerase chain reaction
Vero: Verda Reno
VP1: Viral protein 1
